# High-Throughput Sequencing of Small RNAs from Pollen and Silk and Characterization of miRNAs as Candidate Factors Involved in Pollen-Silk Interactions in Maize

**DOI:** 10.1371/journal.pone.0072852

**Published:** 2013-08-21

**Authors:** Xiao Ming Li, Ya Lin Sang, Xiang Yu Zhao, Xian Sheng Zhang

**Affiliations:** 1 State Key Laboratory of Crop Biology, Shandong Key Laboratory of Crop Biology, College of Life Sciences, Shandong Agricultural University, Tai’an, Shandong, China; 2 College of Forestry, Shandong Agricultural University, Tai’an, Shandong, China; Wuhan University, China

## Abstract

In angiosperms, successful pollen-pistil interactions are the prerequisite and guarantee of subsequent fertilization and seed production. Recent profile analyses have helped elucidate molecular mechanisms underlying these processes at both transcriptomic and proteomic levels, but the involvement of miRNAs in pollen-pistil interactions is still speculative. In this study, we sequenced four small RNA libraries derived from mature pollen, *in vitro* germinated pollen, mature silks, and pollinated silks of maize (*Zea mays* L.). We identified 161 known miRNAs belonging to 27 families and 82 novel miRNAs. Of these, 40 conserved and 16 novel miRNAs showed different expression levels between mature and germinated pollen, and 30 conserved and eight novel miRNAs were differentially expressed between mature and pollinated silks. As candidates for factors associated with pollen-silk (pistil) interactions, expression patterns of the two sets of differentially expressed miRNAs were confirmed by stem-loop real-time RT-PCR. Transcript levels of 22 predicted target genes were also validated using real-time RT-PCR; most of these exhibited expression patterns contrasting with those of their corresponding miRNAs. In addition, GO analysis of target genes of differentially expressed miRNAs revealed that functional categories related to auxin signal transduction and gene expression regulation were overrepresented. These results suggest that miRNA-mediated auxin signal transduction and transcriptional regulation have roles in pollen-silk interactions. The results of our study provide novel information for understanding miRNA regulatory roles in pollen-pistil interactions.

## Introduction

Appropriate pollen-pistil interactions are essential to subsequent successful reproduction and seed formation in angiosperms [1]. At the beginning of the reproductive process, mature pollen grains are released from the dehisced anthers and land on the receptive stigmatic surface. After pollen capture, compatible pollen grains hydrate and germinate to extrude pollen tubes. Elongated by tip growth, pollen tubes penetrate the cell layers of the stigma, navigate within the style tissues, and eventually reach the ovule where fertilization occurs [2]. Incompatible pollen grains, on the other hand, are arrested at certain points in their journey toward the ovule [3]. Pollen-pistil interactions are a series of intensively regulated processes that ensure delivery of appropriate male gametes to female ones [4]. During these processes, pollen controls germination and tube growth, while the pistil provides the foundation and directional guidance for pollen tube navigation [5]. Because pollen-pistil interactions are essential to seed and fruit formation, revealing that their mechanisms is of great importance, both for understanding the completion of the plant life cycle and for enhancing agricultural production. Recent transcriptomic and proteomic studies have extended our knowledge of pollen/pistil gene and protein expression and revealed genes possibly involved in pollen-pistil interactions [2], [6]–[8]. The post-transcriptional regulation underlying these interactions remains largely unknown, however.

MicroRNAs (miRNAs), which are negative regulators of gene expression, are involved in many biological processes in eukaryotic species [9]. Most miRNA genes are transcribed by RNA polymerase II into primary miRNAs (pri-miRNAs), which are converted in turn into single-strand stem-loop precursors (pre-miRNAs) and then miRNA:miRNA* duplexes by proteins such as Dicer like 1, HYPONASTIC LEAVES1, and SERRATE in plants [10]. miRNAs are then incorporated into RNA-induced silencing complexes (RISCs) and cleave their target mRNAs with the assistance of ARGONAUTE proteins, while miRNA*s are usually degraded [11]. To elucidate the post-transcriptional regulation underlying various biological processes, miRNA expression profiles during these processes have recently been analyzed in different plant species [12]–[15]. No studies identifying miRNAs with potential roles in pollen-pistil interactions are available, however.

Maize (*Zea mays* L.) is a model species for studying pollen-pistil interactions, and is one of the most important crops in the world [16]. In this study using maize as the material, we sequenced four small RNA libraries obtained from mature pollen (MP), *in vitro* germinated pollen (GP), mature silks (pistils; MS), and pollinated silks (PS). Two sets of differentially expressed miRNAs were identified and considered as candidate factors related to pollen-silk interactions. The first set included miRNAs differentially expressed during pollen germination and tube growth, and the second set included miRNAs responsive to pollination in silks. GO analysis of their target genes revealed the enrichment of terms related to auxin signaling and transcriptional regulation, suggesting the potential regulation of these processes by miRNAs during pollen-pistil interactions.

## Results and Discussion

### High-throughput sequencing of small RNAs from maize pollen and silk tissues

**Figure 1:**
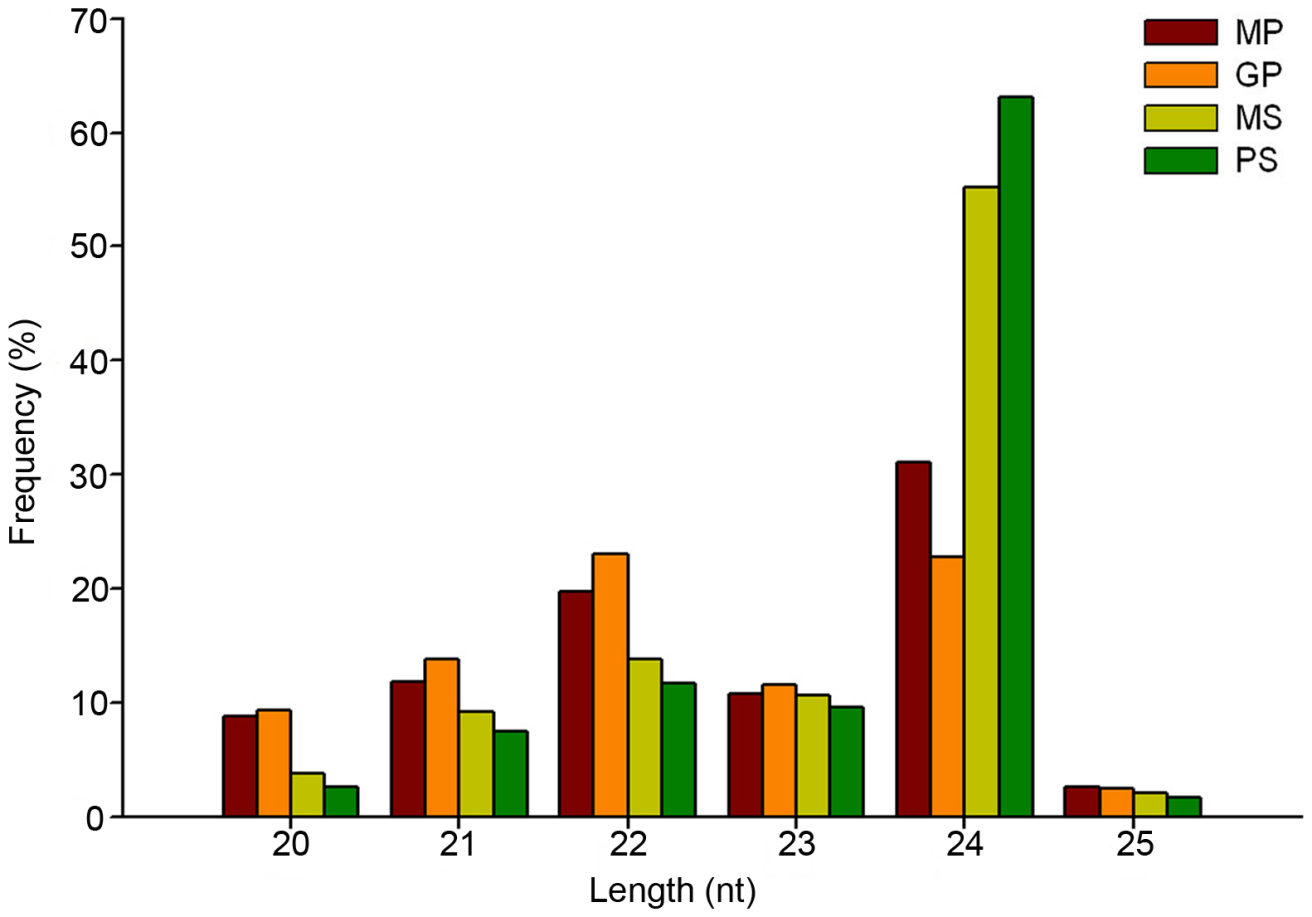
Length distribution of small RNAs identified from mature pollen (MP), germinated pollen (GP), mature silks (MS), and pollinated silks (PS).

To profile small RNAs expressed in maize pollen and silk, and to identify miRNAs potentially involved in regulation of pollen-silk interactions, we sequenced four small RNA libraries from mature pollen (MP), *in vitro* germinated pollen (GP), mature silks (MS), and pollinated silks (PS) using Solexa technology. We obtained 6,433,541, 11,133,002, 9,173,416, and 8,327,428 raw reads from MP, GP, MS, and PS, respectively. After removing adaptors, contaminants, and low-quality sequences, 3,638,941, 10,575,042, 5,775,303, and 5,205,129 high-quality clean reads 18–30-nt long were generated from MP, GP, MS and PS, respectively. Length distribution based on total number of clean reads from the four sequenced libraries is illustrated in Figure 1. Small RNAs with 24 and 22 nt were the two major classes, similar to previous findings in many tissues of maize and rice [13], [14], [17].

The clean reads were mapped to the maize genome (B73 RefGen_2v, released 5a.59) using SOAP, revealing 3,107,037 (85.38%), 9,527,191 (90.09%), 4,935,606 (85.46%), and 4,495,910 (86.37%) perfectly matching sequences from MP, GP, MS, and PS, respectively. A total of 22,065,744 (87.58%) genome-matched sequences, representing 9,859,314 (81.34%) unique sequences, were identified (Table 1). Based on a Blastn search against Rfam base, sequences corresponding to rRNAs, tRNAs, snoRNAs, and other small RNAs were identified (Table 1). A large number of sequences (22,969,291 representing 9,816,635 unique sequences) could not be annotated. The well-represented unannotated small RNAs provided an opportunity to identify novel miRNAs.

**Table 1:** Total genome-matched sequences identified from the four libraries.

	Unique sequences	Percent (%)	Total sequences	Percent (%)
Total Reads	9,859,314	100.00	22,065,744	100.00
rRNA	24,254	0.25	1,345,433	6.10
tRNA	3,106	0.03	143,074	0.65
snoRNA	3,291	0.03	15,050	0.07
Other small RNAs	8,927	0.09	129,767	0.59
Known miRNAs	1,401	0.01	463,129	2.10
Unannotated	9,818,335	99.58	19,969,291	90.50

### Identification of conserved miRNAs

To identify known maize miRNAs, candidate sequences were Blastn-searched against known mature miRNAs and their precursors in miRBase (version 18.0) to recover perfectly matching sequences from maize. We identified 161 conserved miRNAs belonging to 27 miRNA families from the four libraries: 97 in MP, 105 in GP, 143 in MS, and 147 in PS (Table S1). Total read numbers of conserved miRNAs in the four libraries were 94,487 (MP), 168,406 (GP), 643,011 (MS), and 580,305 (PS) (Table S1).

**Figure 2:**
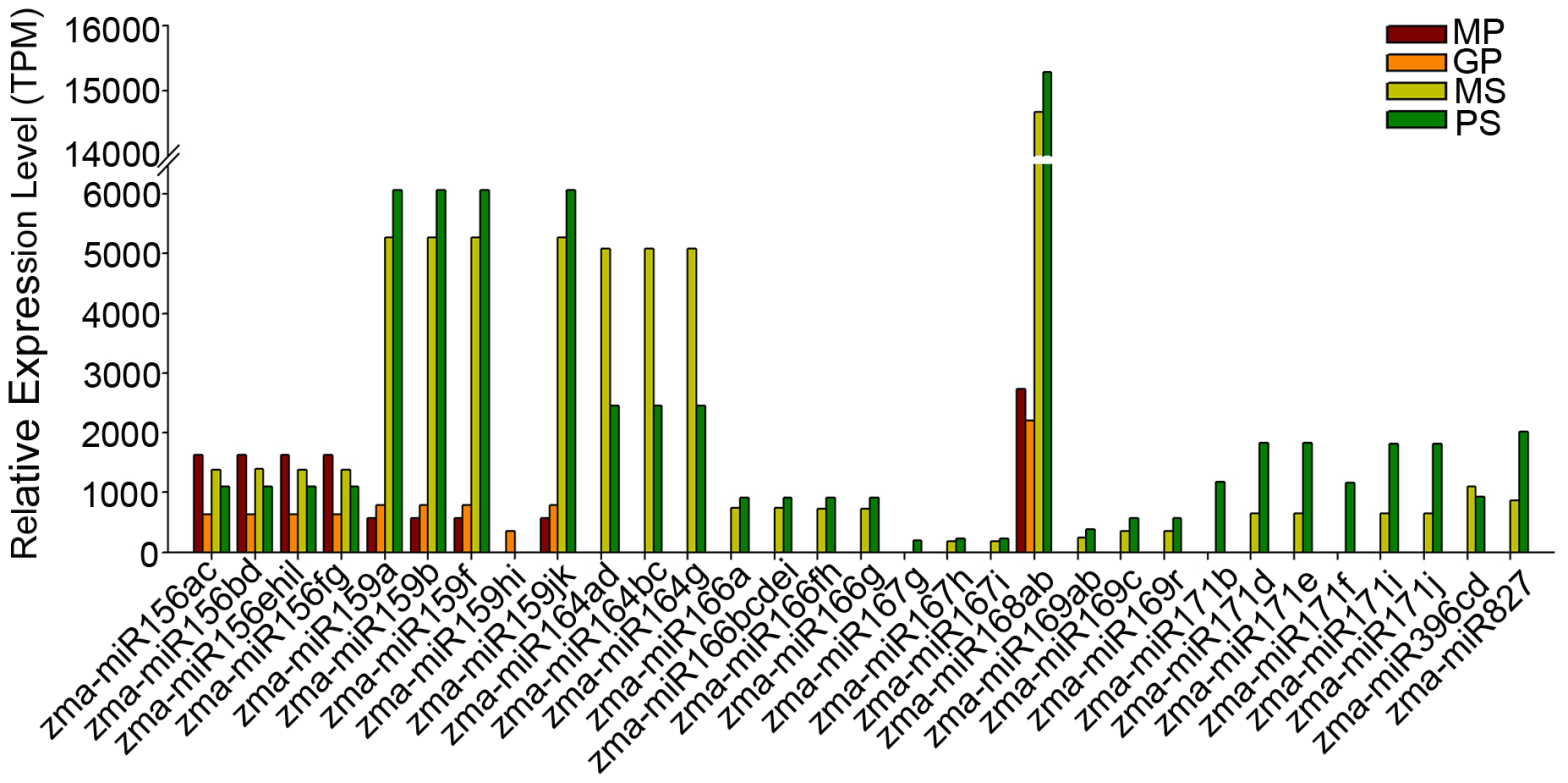
Highly expressed miRNAs in MP, GP, MS, and PS.

We further analyzed conserved miRNAs with relatively high (>1,000) read numbers. As shown in Figure 2, zma-miR156a/b/c/d/e/f/g/h/i/l and miR168a/b were highly abundant in all four libraries. miR156 is involved in diverse processes in various model species, including anther development, juvenile-to-adult phase transition, gibberellin signal transduction, and flower and leaf development [18]–[21]. In maize, however, the accumulation of zma-miR156 in pollen and silk samples suggests its possible role in reproductive processes. miR168 is a known feedback regulator of the miRNA pathway, where it targets *ARGONAUTE1* (*AGO1*), which is important for miRNA function [22], [23]. The modulation of *AGO1* mediated by miR168 is essential to proper development in Arabidopsis [22]. We suggest that miR168-controlled feedback regulation is also required for plant reproduction. Members of several other miRNA families, including zma-miR164, zma-miR167, zma-miR169, zma-miR171, and zma-miR827, were also highly expressed in MS and PS; they were barely detected in MP and GP, however, indicating their specific roles in maize female reproductive tissues.

To further understand the potential biological roles of identified conserved miRNAs, we predicted their target genes using a previously described approach [24]. Based on this method, 144 target genes were identified (Table S3).

### Prediction of novel miRNAs

In plants, miRNAs are excised from the stem of their single-stranded precursors' stem-loop structures [25]. Based on this fundamental defining miRNA characteristic, we predicted novel miRNAs using the following criteria. First, flanking sequences of the unannotated small RNAs were analyzed with the Einverted program of the Emboss software package, and secondary structures were predicted using RNAfold [26]–[28]. Second, MirCheck analysis was applied to filter out sequences mapping to the loop region. Sequences passing MirCheck were further inspected manually [27]. Third, we removed putative miRNAs with minimal folding free energy indexes ≤ 0.85, based on previous studies [29]. Finally, to minimize noise, only small RNAs with more than five reads in at least one library were selected. A total of 82 novel miRNA sequences classifiable into 61 families were identified (Table S2). The star sequences of some miRNAs were also identified. The lengths of these newly identified miRNAs ranged from 20 to 24 nt. Their negative folding free energies varied from −19.9 to −171.2 kcal mol^−1^, with an average of −65 kcal mol^−1^, similar to values uncovered in other recent studies on maize [17], [30]. Unlike conserved miRNAs, novel miRNAs were nearly equally distributed across the four libraries, with 51 in MP, 58 in GP, 69 in MS, and 52 in PS. Target genes of these novel miRNAs were predicted using the same approach employed for the conserved miRNAs; 44 possible targets were identified (Table S4).

For some novel miRNAs, both sequences from 3p and 5p arms were identified (Table S2). Most read counts were similar between the two arms, indicating accumulation of both miRNAs and miRNA*s. Similar observations have been made in many other tissues of maize [14], [17]. It is currently believed that some miRNA*s may be able to accumulate in large quantities and exert the same functions as miRNAs—regulating their target genes—in both plants and animals [31]–[34]. Because such miRNA*s have been referred to as *de facto* miRNAs [14], we considered all the newly identified 3p- and 5p-sequences in our dataset to be novel miRNAs.

### Identification of miRNAs differentially expressed between mature and germinated pollen

Identification and characterization of miRNAs differentially expressed between MP and GP may facilitate our understanding of post-transcriptional regulation during pollen germination and tube growth. For this purpose, we compared normalized expression levels of miRNAs from MP and GP, selecting miRNAs with fold-changes greater than 1.5 (log_2_ ratio) and *P*-values less than 0.01 (Chi^2^ test). As shown in Table 2, 40 conserved and 16 novel miRNAs were found to be differentially expressed between MP and GP.

**Table 2:** miRNAs differentially-expressed between mature and germinated pollen.

miRNA name	Sequence (5′ —3′)	Length	Fold-change	*P*-value
zma-miR159h	UUUGGAGUGAAGGGAGCUCUG	21	1.640	0
zma-miR159i	UUUGGAGUGAAGGGAGCUCUG	21	1.640	0
zma-miR390a	AAGCUCAGGAGGGAUAGCGCC	21	2.256	0
zma-miR390b	AAGCUCAGGAGGGAUAGCGCC	21	2.256	0
zma-miR399b	UGCCAAAGGAGAGCUGUCCUG	21	7.826	0.005147
novel-2-5p	CAAAGAGAAUUGAGGGGGCUA	21	3.389	0
novel-6-3p	AUAUUAAUUAAGUAAUCAUUGA	22	9.765	0
novel-7-3p	AAAUCCUUUGGGAAAAUGAGG	21	3.904	0.000001
novel-14a-5p	AGAGGGGAUUGGAGGGGCUA	20	1.756	0.000101
novel-23-5p	CGGUGUAACACCCUGAAUUUGA	22	8.856	0.000064
novel-27-3p	UCCACUACGUCGGCAAGGGUG	21	2.076	0.003957
novel-29-3p	AUCACCUUCGGUUUUGUGGCU	21	2.548	0
novel-35-5p	CACCAAGUUGGUAAGGUGUUGG	22	2.294	0.001072
novel-41-5p	UUUGGAUUGAAUUGGUUGGUG	21	1.836	0.000465
novel-46-3p	AAUAUUUGAUCUGUUAGAUGGUCU	24	2.354	0.000008
novel-57-5p	AAACACAUAUGUGAUGUAGCGG	22	1.718	0.000005
novel-59-3p	CAAUUUAGGGACUAAAACGAA	21	2.037	0
zma-miR156k	UGACAGAAGAGAGCGAGCAC	20	−1.539	0.008325
zma-miR160a	UGCCUGGCUCCCUGUAUGCCA	21	−3.539	0.000047
zma-miR160b	UGCCUGGCUCCCUGUAUGCCA	21	−3.539	0.000047
zma-miR160c	UGCCUGGCUCCCUGUAUGCCA	21	−3.539	0.000047
zma-miR160d	UGCCUGGCUCCCUGUAUGCCA	21	−3.539	0.000047
zma-miR160e	UGCCUGGCUCCCUGUAUGCCA	21	−3.539	0.000047
zma-miR160g	UGCCUGGCUCCCUGUAUGCCA	21	−3.539	0.000047
zma-miR162	UCGAUAAACCUCUGCAUCCA	20	−3.709	0
zma-miR164a	UGGAGAAGCAGGGCACGUGCA	21	−6.780	0.000461
zma-miR164b	UGGAGAAGCAGGGCACGUGCA	21	−6.780	0.000461
zma-miR164c	UGGAGAAGCAGGGCACGUGCA	21	−6.780	0.000461
zma-miR164d	UGGAGAAGCAGGGCACGUGCA	21	−6.780	0.000461
zma-miR164g	UGGAGAAGCAGGGCACGUGCA	21	−6.780	0.000461
zma-miR166f	UCGGACCAGGCUUCAUUCCC	20	−1.823	0
zma-miR166g	UCGGACCAGGCUUCAUUCCC	20	−1.815	0
zma-miR166h	UCGGACCAGGCUUCAUUCCC	20	−1.823	0
zma-miR166m	UCGGACCAGGCUUCAUUCCUC	21	−1.954	0.00468
zma-miR167a	UGAAGCUGCCAGCAUGAUCUA	21	−2.887	0
zma-miR167b	UGAAGCUGCCAGCAUGAUCUA	21	−3.124	0
zma-miR167c	UGAAGCUGCCAGCAUGAUCUA	21	−2.887	0
zma-miR167d	UGAAGCUGCCAGCAUGAUCUA	21	−2.887	0
zma-miR167e	UGAAGCUGCCAGCAUGAUCUG	21	−3.346	0.000211
zma-miR167f	UGAAGCUGCCAGCAUGAUCUG	21	−3.346	0.000211
zma-miR167g	UGAAGCUGCCAGCAUGAUCUG	21	−4.124	0.000173
zma-miR167h	UGAAGCUGCCAGCAUGAUCUG	21	−4.124	0.000173
zma-miR167i	UGAAGCUGCCAGCAUGAUCUG	21	−4.124	0.000173
zma-miR167j	UGAAGCUGCCAGCAUGAUCUG	21	−3.346	0.000211
zma-miR169a	CAGCCAAGGAUGACUUGCCGA	21	−2.861	0.004008
zma-miR169b	CAGCCAAGGAUGACUUGCCGA	21	−2.861	0.004008
zma-miR396a	UUCCACAGCUUUCUUGAACUG	21	−2.398	0
zma-miR396b	UUCCACAGCUUUCUUGAACUG	21	−2.398	0
zma-miR408	CUGCACUGCCUCUUCCCUGGC	21	−4.346	0.000036
zma-miR408b	CUGCACUGCCUCUUCCCUGGC	21	−4.346	0.000036
zma-miR827	UUAGAUGACCAUCAGCAAACA	21	−5.780	0.013271
zma-miR2118d	UUCCUGAUGCCUCCCAUGCCUA	22	−3.539	0.004014
novel-21-5p	GUCUGCAAGCUUGUUAAGGGGC	22	−4.575	0
novel-26-3p	UCCCCUUCAAUUCCCUCUGGU	21	−4.709	0.000002
novel-38b-3p	AAUCCUCCUCUGGAUUGGUGU	21	−2.539	0.000002
novel-56-5p	UGAAUGGUGGAGCUUGGAGCC	21	−2.460	0

Five conserved and 12 novel miRNAs, including zma-miR159h/i, zma-miR390a/b and novel-a-5p, were up-regulated in GP compared with MP (Table 2). Previous studies have shown that spatial and temporal regulation of miR159a/b/c by APC/C is required for mitotic progression in male gametophytes in Arabidopsis [35]. On the other hand, a *mir159ab* double mutant did not exhibit defects in pollen germination [36]. These results suggest that miR159a/b/c participates in pollen development, but not in pollen germination and tube growth. In our study, different members of the miRNA159 family displayed diverse expression patterns. miR159a/b/c expression levels varied moderately between MP and GP, whereas miR159h/i exhibited significant changes, indicating the probable involvement of miR159h/i in pollen germination and tube growth. These results suggest that individual members of the same miRNA family may execute their respective functions through different expression patterns. Another miRNA up-regulated in GP, miR390a/b, has been shown to respond to the concentration of auxin, a stimulator of pollen tube growth, and to mediate its signal transduction [37], [38]. In addition, novel-a-5p was predicted to target SNARE-associated Golgi proteins involved in vesicle trafficking during pollen tube elongation [39], [40]. It is thus possible that zma-miR390a/b and novel-a-5p participate in regulation of maize pollen tube elongation.

Thirty-five conserved and four novel miRNAs were down-regulated in GP, of which 11 conserved and three novel miRNA sequences were detected with read counts greater than 10 in at least one library (Table 2; Tables S1 and S2). Several of these down-regulated members may be involved in pollen tube growth. For instance, miR167 controls the expression of *ARF6* and *ARF8*, which are essential for pollen tube elongation [41]. Over-expression of *MIR167b* in Arabidopsis leads to defective pollen germination [42]. We suggest that the observed down-regulation of miR167 in GP ensures normal *ARF6* and *ARF8* expression and promotes pollen germination and tube growth. Another miRNA down-regulated in GP, miR166, influences establishment of adaxial/abaxial leaf polarity in maize, and regulates shoot apical meristem and floral development in Arabidopsis [43]–[45]. Finally, miR396 mediates leaf development in Arabidopsis by controlling *GROWTH-REGULATING FACTOR* [46]. In maize, however, the obvious down-regulation of miR166f/g/h and miR396a/b in GP compared with MP implies that their targets are probably involved in pollen tube growth.

### Characterization of miRNAs differentially expressed pre- and post-pollination in silks

Although pollinated silks were harvested after shaking off unadhered pollen grains, PS was still a mixture of pollinated silks, germinated pollen, and a few pollen grains. In spite of their low percentages in PS, residual pollen grains and tubes could interfere with identification of miRNAs differentially expressed between MS and PS before and after pollination. More specifically, miRNAs accumulating at high levels in MP and/or GP might be considered to be up-regulated in PS compared with MS even though their expression levels were unchanged between mature and pollinated silks. In addition, miRNAs varying moderately in silks pre-and post-pollination might be identified as significantly down-regulated in PS because of their lower levels in PS compared with those in MS. To avoid these errors, we used the following criteria to select miRNAs differentially expressed between MS and PS. First, miRNAs displaying expression level increases more than 1.5-fold (log_2_ ratio) higher in PS than in MS, and with more than 10-fold higher read counts in PS or MS than in GP or MP were considered to be up-regulated in PS. Second, miRNAs with more than 2-fold (log_2_ ratio) decreased expression in PS compared with MS were considered to be down-regulated in PS. On this basis, 30 conserved and eight novel miRNAs were identified as differentially expressed in silks pre- and post-pollination (Table 3).

**Table 3:** miRNAs differentially-expressed before and after pollination in silks.

miRNA name	Sequence (5′ —3′)	Length	Fold-change	*P*-value
zma-miR169f	UAGCCAAGGAUGACUUGCCUA	21	3.320	0.00015
zma-miR169g	UAGCCAAGGAUGACUUGCCUA	21	3.320	0.00015
zma-miR169h	UAGCCAAGGAUGACUUGCCUA	21	3.320	0.00015
zma-miR169l	UAGCCAGGGAUGAUUUGCCUG	21	7.264	0.003042
zma-miR171b	UUGAGCCGUGCCAAUAUCAC	20	7.338	0
zma-miR171f	UUGAGCCGUGCCAAUAUCACA	21	11.125	0
zma-miR395a	GUGAAGUGUUUGGGGGAACUC	21	3.089	0
zma-miR395b	GUGAAGUGUUUGGGGGAACUC	21	3.097	0
zma-miR395d	GUGAAGUGUUUGGGGGAACUC	21	3.093	0
zma-miR395e	GUGAAGUGUUUGGGGGAACUC	21	3.089	0
zma-miR395f	GUGAAGUGUUUGGGGGAACUC	21	3.093	0
zma-miR395g	GUGAAGUGUUUGGGGGAACUC	21	3.093	0
zma-miR395h	GUGAAGUGUUUGGGGGAACUC	21	3.089	0
zma-miR395i	GUGAAGUGUUUGGGGGAACUC	21	3.093	0
zma-miR395j	GUGAAGUGUUUGGGGGAACUC	21	3.089	0
zma-miR395n	GUGAAGUGUUUGGGGGAACUC	21	3.093	0
zma-miR395p	GUGAAGUGUUUGGGGGAACUC	21	3.089	0
zma-miR528a	UGGAAGGGGCAUGCAGAGGAG	21	1.609	0.000567
zma-miR528b	UGGAAGGGGCAUGCAGAGGAG	21	1.609	0.000567
novel-9-5p	AAACCAUCUGAUCCGUUAGAUCGU	24	1.887	0.002821
novel-13-3p	AAAACCCCCUGACGCAGCACCGUU	24	3.057	0.000812
novel-15-3p	AGCACCGUUGGAUAUGGAGGGUGU	24	3.237	0
novel-20-5p	GUUCGUUUUGGAGUGGAUUGAGGG	24	1.915	0.005434
novel-33-5p	UAGCCAAGCAUGAUUUGCCCGU	22	1.632	0
zma-miR159h	UUUGGAGUGAAGGGAGCUCUG	21	−2.502	0.000001
zma-miR159i	UUUGGAGUGAAGGGAGCUCUG	21	−2.502	0.000001
zma-miR160a	UGCCUGGCUCCCUGUAUGCCA	21	−2.456	0
zma-miR160b	UGCCUGGCUCCCUGUAUGCCA	21	−2.456	0
zma-miR160c	UGCCUGGCUCCCUGUAUGCCA	21	−2.456	0
zma-miR160d	UGCCUGGCUCCCUGUAUGCCA	21	−2.456	0
zma-miR160e	UGCCUGGCUCCCUGUAUGCCA	21	−2.456	0
zma-miR160g	UGCCUGGCUCCCUGUAUGCCA	21	−2.456	0
zma-miR393a	UCCAAAGGGAUCGCAUUGAUCU	22	−2.309	0
zma-miR393b	UCCAAAGGGAUCGCAUUGAUCC	22	−4.242	0
zma-miR393c	UCCAAAGGGAUCGCAUUGAUCU	22	−2.309	0
novel-19-5p	GUUUGGAGGAGAUUGAGGGGC	21	−7.436	0.002543
novel-56-5p	UGAAUGGUGGAGCUUGGAGCC	21	−7.114	0.006944
novel-57-3p	CCUAGAUGACAUGUGUGUUUUU	22	−7.284	0.004193

The 30 conserved differentially expressed miRNAs belonged to seven families (Table 3). Interestingly, all seven of these miRNA families have been found to respond to stress and/or defense processes [47]–[58]. Five of them—miR169, miR171, miR393, miR395, and miR528—are responsive to drought and/or salt stress [47]–[53]. For example, miR169 over-expression confers enhanced drought tolerance in tomato [54]. In addition, miR159, miR160, and miR171 are involved in resistance to pathogen invasion [55]–[58]. In wheat, miR159 takes part in cellular defense responses to rust fungus [55]. miR160 responds to fungal invasion in poplar, and is associated with resistance to soybean mosaic virus in soybean [56], [57]. Infection of turnip mosaic virus inhibits miR171-guided cleavage of mRNA targets, and induces developmental defects in Arabidopsis [58]. Recent studies have revealed overlaps between pollination and stress responses at both transcriptional and proteomic levels [7], [59], [60]. These findings suggest that the genetics regulating pollination processes are closely connected with those involved in stress responses [60].

Previous studies have found that pollen rehydration processes on dry stigmatc surfaces may cause water deficit stress and alter expression of genes responsive to drought stress [7], [59]. Moreover, pollen tube reception and fungal invasion have been shown to share conserved components, indicating similar molecular mechanisms in these two processes [61]. Consistent with these observations, all conserved miRNAs identified as differentially expressed pre- vs. post-pollination in this study were involved in drought and/or fungal invasion responses. We thus assume that these miRNAs play a role in pollen-silk interactions; however, dissecting the underlying molecular mechanisms requires further investigation.

### Analysis of miRNAs potentially involved in pollen-silk interactions

miRNAs associated with pollen-silk interactions could be derived from either pollen or silk. In this study, we identified miRNAs differentially expressed during *in vitro* pollen germination and tube growth, as well as from silks pre- and post-pollination. Most current information about pollen germination and tube growth has been obtained from *in vitro* studies [62]. Many common cellular and molecular events, as well as genes regulating pollen germination and tube growth, are shared between *in vivo* and *in vitro* processes [8], [62]. We therefore assumed that most of the differentially expressed miRNAs identified from *in vitro* germinated pollen also play a role in *in vivo* processes. It was thus likely that the two sets of differentially expressed miRNAs, including 72 conserved and 24 novel ones, contained miRNAs functioning in pollen-silk interactions (Tables 2 and 3). These differentially expressed miRNAs were subsequently considered as potential factors involved in pollen-silk interactions.

**Figure 3:**
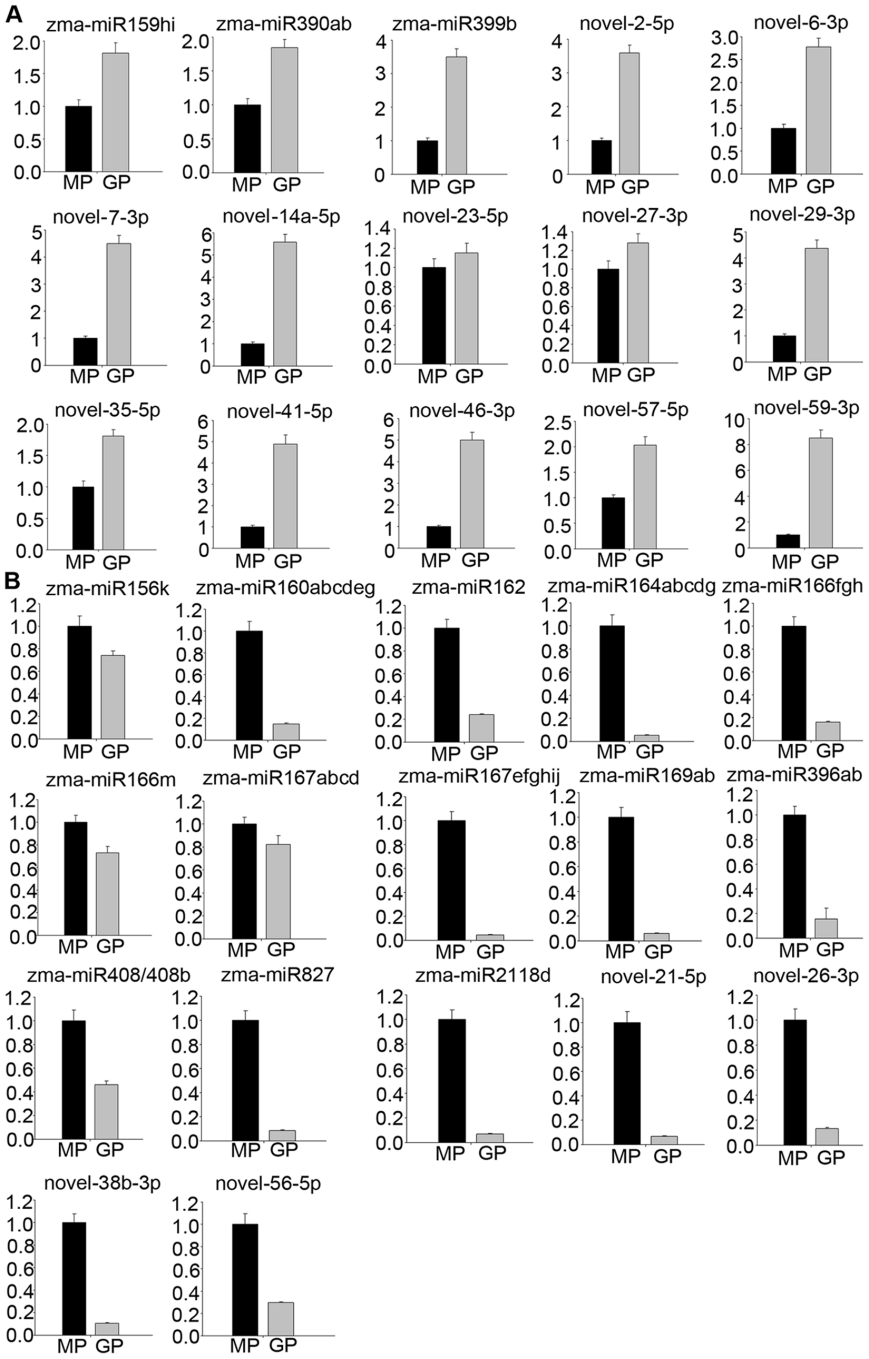
Validation of differentially expressed miRNAs between MP and GP using stem-loop real-time PCR. (A) miRNAs with expression levels up-regulated in GP compared with those of MP. (B) miRNAs down-regulated in GP compared with those of MP. Ordinates indicate relative expression levels.

**Figure 4:**
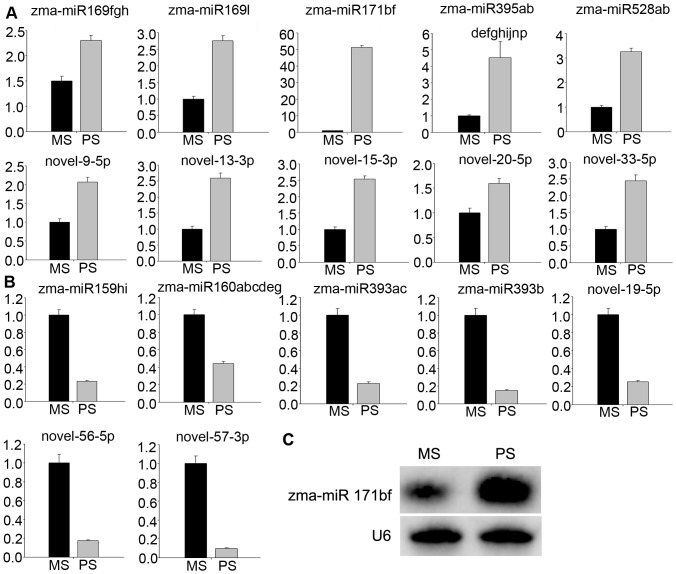
Confirmation of miRNAs differentially expressed between MS and PS using stem-loop real-time PCR. (A) miRNAs with expression levels up-regulated in PS compared with MS. (B) miRNAs down-regulated in PS compared with MS. (C) Northern blot analysis of the expression levels of zma-miR171b/f in MS and PS. Total RNA (20 µg) was loaded for each lane. U6 bands were used as a loading control. For (A) and (B), ordinates indicate relative expression levels.

We used stem-loop real-time RT-PCR to confirm expression patterns of the two sets of differentially expressed miRNAs uncovered via Solexa sequencing. According to our RT-PCR analysis, expression patterns of these differentially expressed miRNAs were all similar to those detected by sequencing (Figures 3, 4A and B). Expression levels of a few members—zma-miR156k, zma-miR166m, zma-miR167a/b/c/d, novel-23-5p, and novel-27-3p—were slightly different (Figure 3). This difference may be due to variations in MP and GP sampling times, or differences in sensitivity and specificity of the two technologies. In general, results obtained by stem-loop real-time RT-PCR were in accordance with the sequencing data. In addition, we further validated the expression level of zma-miR171b/f using northern blot analysis (Figure 4C). The result was consistent with those of stem-loop real-time RT-PCR and sequencing data.

**Figure 5:**
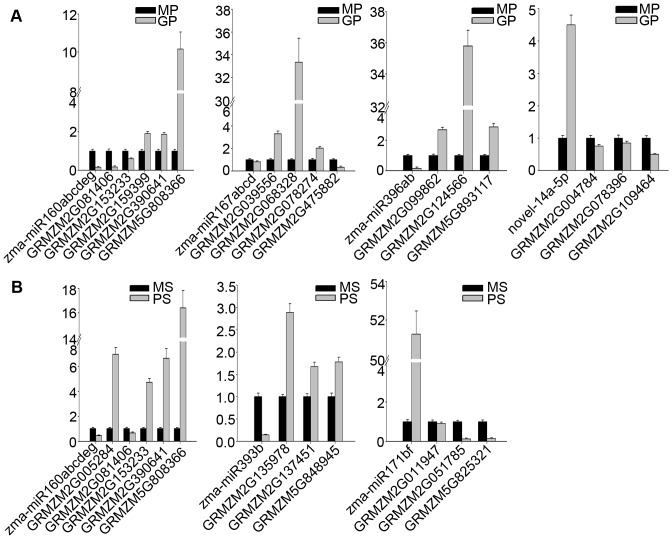
Expression patterns of selected predicted target genes of differentially expressed miRNAs. Expression patterns of putative targets of miRNAs differentially expressed (A) between MP and GP and (B) between MS and PS.

We also analyzed expression of 22 predicted target genes of seven selected miRNAs through real-time RT-PCR. As shown in Figure 5, expressions of most of these putative target genes were negatively correlated with expression of their corresponding miRNAs, indicating that these genes might be repressed by miRNAs through transcriptional regulation. GRMZM2G081406, GRMZM2G153233, and GRMZM2G475882, however, were positively correlated with their miRNAs (Figure 5). It is possible that these three genes were regulated through translational repression by miRNAs.

To investigate their functions and study their potential roles in pollen-silk interactions, we performed Gene Ontology (GO) analysis on predicted target genes of the differentially expressed miRNAs using singular enrichment analysis (SEA) [63]. Six GO terms were found to be enriched (Table 4). Within the GO biological process category, the terms “response to hormone stimulus”, “regulation of gene expression”, and “macromolecule biosynthetic process” were overrepresented (Table 4).

**Table 4:** Overrepresented GO terms of target genes of miRNAs involved in pollen-silk interactions.

GO term	Category^a^	Description	Number in input list^b^	Number in BG/Ref^c^	*P*-value^d^	FDR^e^
GO:0009719	P	Response to hormone stimulus	7	38	5e–12	4.5e–10
GO:0010468	P	Regulation of gene expression	17	2216	5.4e–05	0.0023
GO:0034645	P	Macromolecule biosynthetic process	21	3777	0.00063	0.0072
GO:0003677	F	DNA binding	35	3439	9.1e–11	2e–09
GO:0005634	C	Nucleus	28	2294	7.4e–11	2.5e–09
GO:0043227	C	Membrane-bounded organelle	28	2791	4.3e–09	4.8e–08

a GO categories: P, Biological Process; F, Molecular Function; C, Cellular Component.

b Query item number in target genes.

c Query item number in maize genome version 5a.

d Determined by Fisher's exact test.

e False discovery rate, determined by the Yekutieli procedure.

Notably, all seven genes enriched in the “response to hormone stimulus” category—GRMZM2G005284, GRMZM2G078274, GRMZM2G081406, GRMZM2G153233, GRMZM2G159399, GRMZM2G390641, and GRMZM2G475882—were auxin response factors (Table S3), indicating that this hormone might be involved in pollen-silk interactions. The functions of miR167-regulated *ARF6* and *ARF8* in pollen germination and tube growth have been discussed above. Real-time RT-PCR analysis revealed that the expression level of GRMZM2G078274, which encodes ARF6, was negatively correlated with that of zma-miR167a/b/c/d (Figure 5; Table S3). In addition, zma-miR160a/b/c/d/e/g and zma-miR393a/b/c were observed to be down-regulated in silks after pollination (Figure 4B; Table 3). Recent studies in model species have provided evidence that miR160 targets *ARF10*, *ARF16*, and *ARF17*, and that miR393b targets *TIR1/AFB2*, the gene encoding an auxin receptor [64], [65]. Our real-time RT-PCR results revealed that expression levels of GRMZM2G005284, GRMZM2G159399, GRMZM2G390641, and GRMZM5G808366, which encode ARF10, ARF16, ARF16, and ARF17, respectively, were elevated when zma-miR160a/b/c/d/e/g was down-regulated (Figure 5; Table S3). Additionally, three targets (GRMZM2G135978, GRMZM2G137451, and GRMZM5G848945) encoding auxin receptors exhibited opposite expression patterns to zma-miR393 (Figure 5; Table S3). Observed down-regulation of zma-miR160 and zma-miR393 therefore implies increased accumulation of *ARF10*, *ARF16*, *ARF17*, and *TIR1/AFB2* during pollen-silk interactions, indicating activation of auxin signal transduction. miR393 is suggested to function as an integrator of complex environmental cues through regulation of auxin receptors [66]. We propose that during pollination processes, zma-miR393 responds to signals released from pollen grains and tubes by activating auxin signal transduction, which enables silk cells to achieve suitable states for acceptance and guidance of elongating pollen tubes. Reduced fertility observed in the mutant *miR393b-1* supports the notion that zma-miR393 plays an important role in pollen-silk interactions [66].

Moreover, the terms “regulation of gene expression”, “DNA binding”, and “nucleus” were overrepresented in GO biological processes, molecular functions, and cellular components categories, respectively (Table 4). Taken together, these results indicate that miRNAs may mediate transcriptional regulation through their target genes during pollen-silk interactions.

In conclusion, we obtained 22,065,744 genome-matched small RNA sequences representing 9,859,314 unique sequences from maize pollen and silk samples. Among these sequences, 161 were identified as known miRNAs belonging to 27 families, and 82 were predicted as novel miRNAs. We characterized identified miRNAs differentially expressed between MP and GP, and between MS and PS. Expression patterns of these differentially expressed miRNAs, candidates for involvement in pollen-silk interactions, were confirmed by stem-loop real-time PCR. Regarding miRNAs differentially expressed between MP and GP, several members showed potential involvement in pollen germination or tube growth. In silks, miRNAs responding to pollination have been found in previous studies to be responsive to stresses, especially drought and fungal invasion. GO analysis of putative target genes of the two sets of differentially expressed miRNAs indicated that auxin signal transduction and transcriptional regulation controlled by miRNAs may be involved in the modulation of pollen-silk interactions.

## Materials and Methods

### Plant materials

Maize (*Zea mays* L.) inbred line B73 was used in this study. Plants were grown in the greenhouse at 22°C and 60% relative humidity under a 16h/8h light/dark cycle. Mature pollen (MP) was collected by shaking tassels between 9:00–10:00 a.m. To ensure viability of collected pollen, we tested their *in vitro* germination percentages. Only pollen grains with germination percentages greater than 90% were used. Germinated pollen (GP) was obtained after culturing MP for 30 min in liquid medium [67], with germination percentages greater than 90% obtained (data not shown). Silks extending approximately 5 cm beyond the bracts were harvested as mature silks (MS). To obtain pollinated silks (PS), mature silks were pollinated by an excess of active pollen. After 30 min, most pollen grains had germinated and their tubes had penetrated into silk tissues (data not shown). The pollinated silks were then harvested after shaking off unadhered pollen grains.

### Small RNA library construction and Solexa sequencing

Library construction and sequencing were performed as described previously [68]. Briefly, total RNA was isolated using Trizol reagent (Invitrogen, Carlsbad, CA, USA). Small RNAs ranging from 18–30 nt were gel-purified and ligated to proprietary adaptors at 5′ and 3′ termini. RT-PCR was performed for 18 cycles, and the products were purified and subjected to Solexa sequencing. Sequencing data is available in the GEO database under accession number GSE44787 (http://www.ncbi.nlm.nih.gov/geo/query/acc.cgi?acc=GSE44787).

### Analysis of sequencing data

Low quality reads were produced by an Illumina 1G Genome Analyzer (Illumina, San Diego, CA, USA). Clean reads were obtained after removing adaptor/acceptor sequences, contaminants formed by adaptor-adaptor ligation, and low-quality tags. The clean reads were then mapped to the B73 genome (http://www.maizesequence.org, release 5a.59) using SOAP, and sequences derived from rRNAs, tRNAs, and snoRNAs annotated in the National Center for Biotechnology Information (NCBI) and Rfam database were eliminated [69]. The remaining small RNA sequences were considered as candidates for identification of conserved and novel miRNAs.

### Identification of conserved and novel miRNAs

To identify conserved miRNAs, all candidate sequences were Blastn-searched against miRBase 18.0. Sequences perfectly matched to mature and precursor sequences in the database were identified as conserved miRNAs.

For novel miRNA identification, we used the Einverted program in the Emboss software package to located inverted repeats (stem-loop structures) [26] with the following parameters: threshold  = 30, match score  = 3, mismatch score  = 3, gap penalty  = 6, and maximum repeat length  = 240 [27]. Each inverted repeat was extended 10 nt on either side, and its secondary structure was predicted by RNAfold [28]. Unique reads in inverted repeats were evaluated by MirCheck using default parameters [27]. miRNA sequences passing MirCheck were then inspected manually. Of the remaining sequences, only those with more than five read counts in at least one library and minimal folding free energy indexes (MFEI) higher than 0.85 were selected as novel miRNAs (MFEI =  [(MFE/RNA sequence length) × 100]/(G+C)%) [29].

### Stem-loop real-time PCR

Total RNA was extracted using Trizol reagent (Invitrogen, Carlsbad, CA, USA) and treated by RNase-free DNase I treatment (Promega, Madison, WI, USA) to eliminate genomic DNA contamination. Then, 2 µg total RNA was used for first strand cDNA synthesis with a One Step PrimeScript miRNA cDNA synthesis kit (Takara, Dalian, China). Real-time RT-PCR was carried out with 0.5 µL cDNA on CFX96 real-time system (Bio-Rad, Hercules, CA, USA) using a SYBR *Premix Ex Taq* kit (Takara, Dalian, China). Protocol of the stem-loop real-time PCR was as follows: initial denaturation at 95°C for 10 s, followed by 40 cycles at 95°C for 10 s, 60°C for 30 s. At the end of the PCR reaction, a melting curve was obtained by holding at 95°C for 5 s, cooling to 60°C for 5 s, and then heating slowly at 0.5°C/s until 95°C. Only the results of miRNA amplification that produced one-peak melting curve at the correct annealing temperature were used for next analysis. Simultaneously, the threshold was automatically set and the threshold cycle (Ct value) was recorded. Expression levels of small nuclear RNA U6 were used as a reference for normalization [70]. The ΔΔCt method was used to determine relative expression levels [71]. Four replicates were performed for each experiment. Primers used in real-time PCR analysis are listed in Table S5.

### Northern blot analysis

Northern blot hybridization was performed using the MiRNA Northern Blot Assay Kit (Signosis, Sunnyvale, CA, USA) according to manufacturer's protocol. For each sample, 20 µg total RNA was mixed with RNA loading buffer and load onto one well of 15% polyacrylamide gel. The gel was then run at 60 V until bromophenol blue reaches approximately 3 cm away from the bottom of the gel. RNA was transferred to membrane at 60 V at for l hour using a BioRad Trans-Blot Cell (Bio-Rad, Hercules, CA, USA). Biotin labeled miRNA probe (Signosis, Sunnyvale, CA, USA) was used for hybridization. U6 bands were shown as a loading control.

### Target gene prediction

Potential target genes of miRNAs were predicted using Patscan as previously described [24] with default parameters. Targets were selected according to the following criteria: 1) less than three mismatches between miRNA and target; 2) no insertions and deletions; and 3) no mismatches in positions 10 and 11 of mature miRNAs.

### Validation of predicted target genes by real-time RT-PCR

Total RNA from MP, GP, MS and PS was isolated as described above, and 2 µg total RNA was reverse-transcribed using oligo (dT) primer and M-MLV reverse transcriptase (Promega, Madison, WI, USA) according to manufacturer's protocol. The real-time PCR was performed in triplicate on a CFX96 real-time system (Bio-Rad, Hercules, CA, USA) using SYBR Green Real-time PCR Master Mix (Toyobo, Osaka, Japan). Briefly, 1 µL cDNA was amplified with the following protocol: 94°C for 5 min, followed by 41 cycles of 94°C for 15 s, 65°C for 30 s. Finally, the melting curve was adjusted as 94°C for 5 s and 60°C for 1 min, and then rising to 94°C by increment of 0.5°C/s. 18S rRNA was used for each sample as an internal control. Relative expression levels of the genes were calculated using the ΔΔCt method [71]. All primer sequences were listed in Table S5.

### Identification of differentially expressed miRNAs

Transcripts per million reads (TPM) were used to represent normalized miRNA expression levels. TPM of miRNAs in the four libraries were calculated as: actual miRNA count/total count of clean reads × 1,000,000. For the identification of miRNAs differentially expressed between mature and germinated pollen, we used fold-change  = log_2_ × (GP/MP). Only miRNAs with fold-changes greater than 1.5 and *P*-values less than 0.01 were selected (Chi^2^ test) [72]. For the identification of miRNAs differentially expressed before and after pollination in silks, we used fold-change  = log_2_×(PS/MS). miRNAs with expression level increases 1.5-fold higher in PS, and read counts more than 10-fold greater in PS or MS than in GP and MP were considered to be up-regulated in PS. miRNAs with expression level decreases 2-fold greater in PS compared with MS were considered to be down-regulated in PS.

### Gene Ontology (GO) analysis

We performed GO analysis on target genes of miRNAs involved in pollen-silk interactions using Singular enrichment analysis (SEA) [63] (http://bioinfo.cau.edu.cn/agriGO/analysis.php). Overrepresented gene terms in biological processes, molecular functions, and cellular components categories were selected using the thresholds of *P*-value <0.001 and false discovery rate (FDR) <0.05.

## Supporting Information

Table S1 **List of known miRNAs identified in this study.**
(XLS)

Table S2 **Novel miRNAs predicted in this study.**
(XLS)

Table S3 **Targets of all known miRNAs.**
(XLS)

Table S4 **Targets of all novel miRNAs.**
(XLS)

Table S5 **The primers used in this study.**
(DOC)

## References

[1] Cheung AY (1995) Pollen-pistil interactions in compatible pollination. Proc Natl Acad Sci U S A 92: 3077–3080. 7724518 10.1073/pnas.92.8.3077 PMC42107

[2] Hiscock SJ, Allen AM (2008) Diverse cell signalling pathways regulate pollen-stigma interactions: the search for consensus. New Phytol 179: 286–317. 19086285 10.1111/j.1469-8137.2008.02457.x

[3] Takayama S, Isogai A (2005) Self-incompatibility in plants. Annu Rev Plant Biol 56: 467–489. 15862104 10.1146/annurev.arplant.56.032604.144249

[4] Edlund AF, Swanson R, Preuss D (2004) Pollen and stigma structure and function: the role of diversity in pollination. Plant Cell 16 Suppl: S84–97 10.1105/tpc.015800 PMC2643401 15075396

[5] Sanchez AM, Bosch M, Bots M, Nieuwland J, Feron R, et al (2004) Pistil factors controlling pollination. Plant Cell 16 Suppl: S98–106 10.1105/tpc.017806 PMC2643392 15010514

[6] Xu XH, Chen H, Sang YL, Wang F, Ma JP, et al (2012) Identification of genes specifically or preferentially expressed in maize silk reveals similarity and diversity in transcript abundance of different dry stigmas. BMC Genomics 13: 294. 22748054 10.1186/1471-2164-13-294 PMC3416702

[7] Sang YL, Xu M, Ma FF, Chen H, Xu XH, et al (2012) Comparative proteomic analysis reveals similar and distinct features of proteins in dry and wet stigmas. Proteomics 12: 1983–1998. 22623354 10.1002/pmic.201100407

[8] Qin Y, Leydon AR, Manziello A, Pandey R, Mount D, et al (2009) Penetration of the stigma and style elicits a novel transcriptome in pollen tubes, pointing to genes critical for growth in a pistil. PLoS Genet 5: e1000621. 19714218 10.1371/journal.pgen.1000621 PMC2726614

[9] Bushati N, Cohen SM (2007) microRNA functions. Annu Rev Cell Dev Biol 23: 175–205. 17506695 10.1146/annurev.cellbio.23.090506.123406

[10] Voinnet O (2009) Origin, biogenesis, and activity of plant microRNAs. Cell 136: 669–687. 19239888 10.1016/j.cell.2009.01.046

[11] Jones-Rhoades MW, Bartel DP, Bartel B (2006) MicroRNAS and their regulatory roles in plants. Annu Rev Plant Biol 57: 19–53. 16669754 10.1146/annurev.arplant.57.032905.105218

[12] Peng T, Sun H, Du Y, Zhang J, Li J, et al (2013) Characterization and Expression Patterns of microRNAs Involved in Rice Grain Filling. PLoS One 8: e54148. 23365650 10.1371/journal.pone.0054148 PMC3554753

[13] Wei LQ, Yan LF, Wang T (2011) Deep sequencing on genome-wide scale reveals the unique composition and expression patterns of microRNAs in developing pollen of *Oryza sativa* . Genome Biol 12: R53. 21679406 10.1186/gb-2011-12-6-r53 PMC3218841

[14] Kang M, Zhao Q, Zhu D, Yu J (2012) Characterization of microRNAs expression during maize seed development. BMC Genomics 13: 360. 22853295 10.1186/1471-2164-13-360 PMC3468377

[15] Yang J, Liu X, Xu B, Zhao N, Yang X, et al (2013) Identification of miRNAs and their targets using high-throughput sequencing and degradome analysis in cytoplasmic male-sterile and its maintainer fertile lines of *brassica juncea* . BMC Genomics 14: 9. 23324572 10.1186/1471-2164-14-9 PMC3553062

[16] Dresselhaus T, Lausser A, Marton ML (2011) Using maize as a model to study pollen tube growth and guidance, cross-incompatibility and sperm delivery in grasses. Ann Bot 108: 727–737. 21345919 10.1093/aob/mcr017 PMC3170146

[17] Zhao M, Tai H, Sun S, Zhang F, Xu Y, et al (2012) Cloning and characterization of maize miRNAs involved in responses to nitrogen deficiency. PLoS One 7: e29669. 22235323 10.1371/journal.pone.0029669 PMC3250470

[18] Xing S, Salinas M, Hohmann S, Berndtgen R, Huijser P (2010) miR156-targeted and nontargeted SBP-box transcription factors act in concert to secure male fertility in *Arabidopsis* . Plant Cell 22: 3935–3950. 21177480 10.1105/tpc.110.079343 PMC3027167

[19] Wang JW, Czech B, Weigel D (2009) miR156-regulated SPL transcription factors define an endogenous flowering pathway in *Arabidopsis thaliana* . Cell 138: 738–749. 19703399 10.1016/j.cell.2009.06.014

[20] Yu S, Galvao VC, Zhang YC, Horrer D, Zhang TQ, et al (2012) Gibberellin regulates the *Arabidopsis* floral transition through miR156-targeted SQUAMOSA promoter binding-like transcription factors. Plant Cell 24: 3320–3332. 22942378 10.1105/tpc.112.101014 PMC3462634

[21] Xie K, Wu C, Xiong L (2006) Genomic organization, differential expression, and interaction of SQUAMOSA promoter-binding-like transcription factors and microRNA156 in rice. Plant Physiol 142: 280–293. 16861571 10.1104/pp.106.084475 PMC1557610

[22] Vaucheret H, Vazquez F, Crete P, Bartel DP (2004) The action of ARGONAUTE1 in the miRNA pathway and its regulation by the miRNA pathway are crucial for plant development. Genes Dev 18: 1187–1197. 15131082 10.1101/gad.1201404 PMC415643

[23] Vaucheret H, Mallory AC, Bartel DP (2006) AGO1 homeostasis entails coexpression of *MIR168* and *AGO1* and preferential stabilization of miR168 by AGO1. Mol Cell 22: 129–136. 16600876 10.1016/j.molcel.2006.03.011 PMC2323247

[24] Schwab R, Palatnik JF, Riester M, Schommer C, Schmid M, et al (2005) Specific effects of microRNAs on the plant transcriptome. Dev Cell 8: 517–527. 15809034 10.1016/j.devcel.2005.01.018

[25] Meyers BC, Axtell MJ, Bartel B, Bartel DP, Baulcombe D, et al (2008) Criteria for annotation of plant MicroRNAs. Plant Cell 20: 3186–3190. 19074682 10.1105/tpc.108.064311 PMC2630443

[26] Rice P, Longden I, Bleasby A (2000) EMBOSS: the European Molecular Biology Open Software Suite. Trends Genet 16: 276–277. 10827456 10.1016/s0168-9525(00)02024-2

[27] Jones-Rhoades MW, Bartel DP (2004) Computational identification of plant microRNAs and their targets, including a stress-induced miRNA. Mol Cell 14: 787–799. 15200956 10.1016/j.molcel.2004.05.027

[28] Gruber AR, Lorenz R, Bernhart SH, Neubock R, Hofacker IL (2008) The Vienna RNA websuite. Nucleic Acids Res 36: W70–74. 18424795 10.1093/nar/gkn188 PMC2447809

[29] Zhang BH, Pan XP, Cox SB, Cobb GP, Anderson TA (2006) Evidence that miRNAs are different from other RNAs. Cell Mol Life Sci 63: 246–254. 16395542 10.1007/s00018-005-5467-7 PMC11136112

[30] Liu P, Yan K, Lei YX, Xu R, Zhang YM, et al (2012) Transcript profiling of microRNAs during the early development of the maize brace root via Solexa sequencing. Genomics 101: 149–156.23147674

[31] Okamura K, Phillips MD, Tyler DM, Duan H, Chou YT, et al (2008) The regulatory activity of microRNA* species has substantial influence on microRNA and 3′ UTR evolution. Nat Struct Mol Biol 15: 354–363. 18376413 10.1038/nsmb.1409 PMC2698667

[32] Rajagopalan R, Vaucheret H, Trejo J, Bartel DP (2006) A diverse and evolutionarily fluid set of microRNAs in *Arabidopsis thaliana* . Genes Dev 20: 3407–3425. 17182867 10.1101/gad.1476406 PMC1698448

[33] Yang JS, Phillips MD, Betel D, Mu P, Ventura A, et al (2011) Widespread regulatory activity of vertebrate microRNA* species. RNA 17: 312–326. 21177881 10.1261/rna.2537911 PMC3022280

[34] Griffiths-Jones S, Hui JH, Marco A, Ronshaugen M (2011) MicroRNA evolution by arm switching. EMBO Rep 12: 172–177. 21212805 10.1038/embor.2010.191 PMC3049427

[35] Zheng B, Chen X, McCormick S (2011) The anaphase-promoting complex is a dual integrator that regulates both MicroRNA-mediated transcriptional regulation of *cyclin B1* and degradation of Cyclin B1 during *Arabidopsis* male gametophyte development. Plant Cell 23: 1033–1046. 21441434 10.1105/tpc.111.083980 PMC3082252

[36] Allen RS, Li J, Alonso-Peral MM, White RG, Gubler F, et al (2010) MicroR159 regulation of most conserved targets in *Arabidopsis* has negligible phenotypic effects. Silence 1: 18. 21029441 10.1186/1758-907X-1-18 PMC2988730

[37] Yoon EK, Yang JH, Lim J, Kim SH, Kim SK, et al (2010) Auxin regulation of the *microRNA390*-dependent transacting small interfering RNA pathway in *Arabidopsis* lateral root development. Nucleic Acids Res 38: 1382–1391. 19969544 10.1093/nar/gkp1128 PMC2831332

[38] Wu JZ, Lin Y, Zhang XL, Pang DW, Zhao J (2008) IAA stimulates pollen tube growth and mediates the modification of its wall composition and structure in *Torenia fournieri* . J Exp Bot 59: 2529–2543. 18544613 10.1093/jxb/ern119 PMC2423660

[39] Kato N, He H, Steger AP (2010) A systems model of vesicle trafficking in Arabidopsis pollen tubes. Plant Physiol 152: 590–601. 19933386 10.1104/pp.109.148700 PMC2815877

[40] Guo F, McCubbin AG (2012) The pollen-specific R-SNARE/longin PiVAMP726 mediates fusion of endo- and exocytic compartments in pollen tube tip growth. J Exp Bot 63: 3083–3095. 22345643 10.1093/jxb/ers023 PMC3350921

[41] Wu MF, Tian Q, Reed JW (2006) Arabidopsis *microRNA167* controls patterns of *ARF6* and *ARF8* expression, and regulates both female and male reproduction. Development 133: 4211–4218. 17021043 10.1242/dev.02602

[42] Ru P, Xu L, Ma H, Huang H (2006) Plant fertility defects induced by the enhanced expression of microRNA167. Cell Res 16: 457–465. 16699541 10.1038/sj.cr.7310057

[43] Nogueira FT, Madi S, Chitwood DH, Juarez MT, Timmermans MC (2007) Two small regulatory RNAs establish opposing fates of a developmental axis. Genes Dev 21: 750–755. 17403777 10.1101/gad.1528607 PMC1838527

[44] Jung JH, Park CM (2007) *MIR166*/*165* genes exhibit dynamic expression patterns in regulating shoot apical meristem and floral development in *Arabidopsis* . Planta 225: 1327–1338. 17109148 10.1007/s00425-006-0439-1

[45] Zhu H, Hu F, Wang R, Zhou X, Sze SH, et al (2011) *Arabidopsis* Argonaute10 specifically sequesters miR166/165 to regulate shoot apical meristem development. Cell 145: 242–256. 21496644 10.1016/j.cell.2011.03.024 PMC4124879

[46] Wang L, Gu X, Xu D, Wang W, Wang H, et al (2011) miR396-targeted AtGRF transcription factors are required for coordination of cell division and differentiation during leaf development in *Arabidopsis* . J Exp Bot 62: 761–773. 21036927 10.1093/jxb/erq307 PMC3003814

[47] Zhao B, Ge L, Liang R, Li W, Ruan K, et al (2009) Members of miR-169 family are induced by high salinity and transiently inhibit the NF-YA transcription factor. BMC Mol Biol 10: 29. 19351418 10.1186/1471-2199-10-29 PMC2670843

[48] Li WX, Oono Y, Zhu J, He XJ, Wu JM, et al (2008) The *Arabidopsis* NFYA5 transcription factor is regulated transcriptionally and posttranscriptionally to promote drought resistance. Plant Cell 20: 2238–2251. 18682547 10.1105/tpc.108.059444 PMC2553615

[49] Hwang EW, Shin SJ, Yu BK, Byun MO, Kwon HB (2011) miR171 Family Members are Involved in Drought Response in *Solanum tuberosum* . J Plant Biol 54: 43–48.

[50] Kantar M, Lucas SJ, Budak H (2011) miRNA expression patterns of *Triticum dicoccoides* in response to shock drought stress. Planta 233: 471–484. 21069383 10.1007/s00425-010-1309-4

[51] Xia K, Wang R, Ou X, Fang Z, Tian C, et al (2012) *OsTIR1* and *OsAFB2* downregulation via *OsmiR393* overexpression leads to more tillers, early flowering and less tolerance to salt and drought in rice. PLoS One 7: e30039. 22253868 10.1371/journal.pone.0030039 PMC3254625

[52] Frazier TP, Sun G, Burklew CE, Zhang B (2011) Salt and drought stresses induce the aberrant expression of microRNA genes in tobacco. Mol Biotechnol 49: 159–165. 21359858 10.1007/s12033-011-9387-5

[53] Ferreira TH, Gentile A, Vilela RD, Costa GG, Dias LI, et al (2012) microRNAs associated with drought response in the bioenergy crop sugarcane (*Saccharum* spp.). PLoS One 7: e46703. 23071617 10.1371/journal.pone.0046703 PMC3469577

[54] Zhang X, Zou Z, Gong P, Zhang J, Ziaf K, et al (2011) Over-expression of microRNA169 confers enhanced drought tolerance to tomato. Biotechnol Lett 33: 403–409. 20960221 10.1007/s10529-010-0436-0

[55] Gupta OP, Permar V, Koundal V, Singh UD, Praveen S (2012) MicroRNA regulated defense responses in *Triticum aestivum* L. during *Puccinia graminis* f.sp. *tritici* infection. Mol Biol Rep 39: 817–824. 21633895 10.1007/s11033-011-0803-5

[56] Zhao JP, Jiang XL, Zhang BY, Su XH (2012) Involvement of microRNA-mediated gene expression regulation in the pathological development of stem canker disease in *Populus trichocarpa* . PLoS One 7: e44968. 23028709 10.1371/journal.pone.0044968 PMC3445618

[57] Yin X, Wang J, Cheng H, Wang X, Yu D (2012) Detection and evolutionary analysis of soybean miRNAs responsive to soybean mosaic virus. Planta. 10.1007/s00425-012-1835-3 23328897

[58] Kasschau KD, Xie Z, Allen E, Llave C, Chapman EJ, et al (2003) P1/HC-Pro, a viral suppressor of RNA silencing, interferes with *Arabidopsis* development and miRNA unction. Dev Cell 4: 205–217. 12586064 10.1016/s1534-5807(03)00025-x

[59] Lan L, Li M, Lai Y, Xu W, Kong Z, et al (2005) Microarray analysis reveals similarities and variations in genetic programs controlling pollination/fertilization and stress responses in rice (*Oryza sativa* L.). Plant Mol Biol 59: 151–164. 16217609 10.1007/s11103-005-3958-4

[60] Quiapim AC, Brito MS, Bernardes LA, Dasilva I, Malavazi I, et al (2009) Analysis of the *Nicotiana tabacum* stigma/style transcriptome reveals gene expression differences between wet and dry stigma species. Plant Physiol 149: 1211–1230. 19052150 10.1104/pp.108.131573 PMC2649396

[61] Kessler SA, Shimosato-Asano H, Keinath NF, Wuest SE, Ingram G, et al (2010) Conserved molecular components for pollen tube reception and fungal invasion. Science 330: 968–971. 21071669 10.1126/science.1195211

[62] Dawkins MD, Owens JN (1993) In Vitro and in Vivo Pollen Hydration, Germination, and Pollen-Tube Growth in White Spruce, Picea Glauca (Moench) Voss. Int J Plant Sci 154: 506–521.

[63] Du Z, Zhou X, Ling Y, Zhang Z, Su Z (2010) agriGO: a GO analysis toolkit for the agricultural community. Nucleic Acids Res 38: W64–70. 20435677 10.1093/nar/gkq310 PMC2896167

[64] Liu X, Huang J, Wang Y, Khanna K, Xie Z, et al (2010) The role of *floral organs in carpels*, an Arabidopsis loss-of-function mutation in *MicroRNA160a*, in organogenesis and the mechanism regulating its expression. Plant J 62: 416–428. 20136729 10.1111/j.1365-313X.2010.04164.x

[65] Si-Ammour A, Windels D, Arn-Bouldoires E, Kutter C, Ailhas J, et al (2011) miR393 and secondary siRNAs regulate expression of the *TIR1/AFB2* auxin receptor clade and auxin-related development of Arabidopsis leaves. Plant Physiol 157: 683–691. 21828251 10.1104/pp.111.180083 PMC3192580

[66] Windels D, Vazquez F (2011) miR393: integrator of environmental cues in auxin signaling? Plant Signal Behav 6: 1672–1675. 22067993 10.4161/psb.6.11.17900 PMC3329333

[67] Schreiber DN, Bantin J, Dresselhaus T (2004) The MADS box transcription factor ZmMADS2 is required for anther and pollen maturation in maize and accumulates in apoptotic bodies during anther dehiscence. Plant Physiol 134: 1069–1079. 15001699 10.1104/pp.103.030577 PMC389931

[68] Liu S, Li D, Li Q, Zhao P, Xiang Z, et al (2010) MicroRNAs of *Bombyx mori* identified by Solexa sequencing. BMC Genomics 11: 148. 20199675 10.1186/1471-2164-11-148 PMC2838851

[69] Gardner PP, Daub J, Tate JG, Nawrocki EP, Kolbe DL, et al (2009) Rfam: updates to the RNA families database. Nucleic Acids Res 37: D136–D140. 18953034 10.1093/nar/gkn766 PMC2686503

[70] Wang T, Chen L, Zhao M, Tian Q, Zhang WH (2011) Identification of drought-responsive microRNAs in *Medicago truncatula* by genome-wide high-throughput sequencing. BMC Genomics 12: 367. 21762498 10.1186/1471-2164-12-367 PMC3160423

[71] Livak KJ, Schmittgen TD (2001) Analysis of relative gene expression data using real-time quantitative PCR and the 2(−ΔΔC(T)) Method. Methods 25: 402–408. 11846609 10.1006/meth.2001.1262

[72] Romualdi C, Bortoluzzi S, D′Alessi F, Danieli GA (2003) IDEG6: a web tool for detection of differentially expressed genes in multiple tag sampling experiments. Physiol Genomics. 12: 159–62. 10.1152/physiolgenomics.00096.2002 12429865

