# Supplementary material for: High-Throughput Sequencing of Small RNAs from Pollen and Silk and Characterization of miRNAs as Candidate Factors Involved in Pollen-Silk Interactions in Maize
Source: PLoS One. 2013 Aug 21;8(8):e72852. doi: 10.1371/journal.pone.0072852 (PMC3749131; doi:10.1371/journal.pone.0072852)
Supplement: Table S5 — The primers used in this study. (DOC) [file pone.0072852.s005.doc]

Primers used in stem-loop real-time PCR.

| miRNA name | Sequence | Length |
| --- | --- | --- |
| zma-miR156k | TGACAGAAGAGAGCGAGCAC | 20 |
| zma-miR159hi | TTTGGAGTGAAGGGAGCTCTG | 21 |
| zma-miR160abcdeg | TGCCTGGCTCCCTGTATGCCA | 21 |
| zma-miR162 | GGTCGATAAACCTCTGCATCC | 21 |
| zma-miR164abcdg | TGGAGAAGCAGGGCACGTGCA | 21 |
| zma-miR166fgh | TCGGACCAGGCTTCATTCCC | 20 |
| zma-miR166m | TCGGACCAGGCTTCATTCCTC | 21 |
| zma-miR167abcd | TGAAGCTGCCAGCATGATCTA | 21 |
| zma-miR167efghij | TGAAGCTGCCAGCATGATCTG | 21 |
| zma-miR169ab | CAGCCAAGGATGACTTGCCGA | 21 |
| zma-miR169fgh | TAGCCAAGGATGACTTGCCTA | 21 |
| zma-miR169l | TAGCCAGGGATGATTTGCCTG | 21 |
| zma-miR171bf | CGATTGAGCCGTGCCAATATC | 21 |
| zma-miR390a b | AAGCTCAGGAGGGATAGCG | 19 |
| zma-miR393ac | TCCAAAGGGATCGCATTGATC | 21 |
| zma-miR393b | TCCAAAGGGATCGCATTGATCC | 22 |
| zma-miR395abdefghijnp | GTGAAGTGTTTGGGGGAACTC | 21 |
| zma-miR396ab | TTCCACAGCTTTCTTGAACTG | 21 |
| zma-miR399b | TGCCAAAGGAGAGCTGTCC | 19 |
| zma-miR408/408b | CTGCACTGCCTCTTCCCTGGC | 21 |
| zma-miR528ab | TGGAAGGGGCATGCAGAGGAG | 21 |
| zma-miR827 | TTAGATGACCATCAGCAAACA | 21 |
| zma-miR2118d | TATTCCTGATGCCTCCCATGCC | 22 |
| novel-2-5p | CAAAGAGAATTGAGGGGGCTA | 21 |
| novel-6-3p | ATATTAATTAAGTAATCATTGA | 22 |
| novel-7-3p | AAATCCTTTGGGAAAATGAGG | 21 |
| novel-9-5p | AAACCATCTGATCCGTTAGATCGT | 24 |
| novel-13-3p | AAAACCCCCTGACGCAGCACCGTTA | 25 |
| novel-14a-5p | AGAGGGGATTGGAGGGGCTA | 20 |
| novel-15-3p | AGCACCGTTGGATATGGAGGGTGT | 24 |
| novel-19-5p | GTTTGGAGGAGATTGAGGGGC | 21 |
| novel-20-5p | GTTCGTTTTGGAGTGGATTGAGGG | 24 |
| novel-21-5p | GTCTGCAAGCTTGTTAAGGGGC | 22 |
| novel-23-5p | CGGTGTAACACCCTGAATTTGA | 22 |
| novel-26-3p | TCCCCTTCAATTCCCTCTGGT | 21 |
| novel-27-3p | TCCACTACGTCGGCAAGGGTG | 21 |
| novel-29-3p | ATCACCTTCGGTTTTGTGGC | 20 |
| novel-33-5p | TAGCCAAGCATGATTTGCCCG | 21 |
| novel-35-5p | CACCAAGTTGGTAAGGTGTTGG | 22 |
| novel-38b-3p | AATCCTCCTCTGGATTGGTGT | 21 |
| novel-41-5p | TTTGGATTGAATTGGTTGGTG | 21 |
| novel-46-3p | AATATTTGATCTGTTAGATGGTCT | 24 |
| novel-56-5p | GAATGGTGGAGCTTGGAG | 18 |
| novel-57-3p | CGGCCTAGATGACATGTGTGTTTTTC | 26 |
| novel-57-5p | AAACACATATGTGATGTAGCGG | 22 |
| novel-59-3p | CAATTTAGGGACTAAAACGAAGG | 23 |
| Zm-U6 | ACACGCACAAATCGAGAAATGGTCC | 25 |

**Primers used in real-time PCR analysis of target genes.**

| Target name | Sequence | Length |
| --- | --- | --- |
| GRMZM2G004784-L | AAGCAGCGTAGAACACAA | 18 |
| GRMZM2G004784-R | AAGCAGGAAGCGAGATAAG | 19 |
| GRMZM2G005284-L | CTAGCACAAGAAGACAAG | 18 |
| GRMZM2G005284-R | CCTCCTGATTAGTGATTAG | 19 |
| GRMZM2G011947-L | TTCGCTAGGACTAATGAT | 18 |
| GRMZM2G011947-R | CACACTAATAAGCAGACA | 18 |
| GRMZM2G039556-L | TTATCTGCCTGATGACTTG | 19 |
| GRMZM2G039556-R | GGATTCGTGTATGCTTCA | 18 |
| GRMZM2G051785-L | GACTCCAACTCTACCGAATC | 20 |
| GRMZM2G051785-R | ATACAACGATGCCAACCTT | 19 |
| GRMZM2G068328-L | GCTACATAATCTGGACAAGGT | 21 |
| GRMZM2G068328-R | TGGCACTGAGACACATTC | 18 |
| GRMZM2G078274-L | AGTGGAAGTTCAGGCATAT | 19 |
| GRMZM2G078274-R | GATAACGCATCACATCAAGA | 20 |
| GRMZM2G078396-L | GATGAATCCTCGTATTGG | 18 |
| GRMZM2G078396-R | CTCCATAATGTCTTCTCTG | 19 |
| GRMZM2G081406-L | GGTACTGGTACTGTTAGACT | 20 |
| GRMZM2G081406-R | TCAGACTTGGAACGGATT | 18 |
| GRMZM2G099862-L | GAGAATCACACGAGTAAC | 18 |
| GRMZM2G099862-R | CACAGGTAAGAAGGAAAG | 18 |
| GRMZM2G109464-L | GACAAGAGTGAACGGTAA | 18 |
| GRMZM2G109464-R | GGAGGATGAGTTGATAATGA | 20 |
| GRMZM2G124566-L | GCTCCAATACCTTGTGTT | 18 |
| GRMZM2G124566-R | GCAAGACATTCATTCATTCG | 20 |
| GRMZM2G135978-L | GAGAATGAGATTGAGGATTG | 20 |
| GRMZM2G135978-R | CTTGAGAGTCTTGAGGTT | 18 |
| GRMZM2G137451-L | GTATTCAGCAGTTGTTGGAT | 20 |
| GRMZM2G137451-R | CCTCAGGAGCCTAGATAAC | 19 |
| GRMZM2G153233-L | ATCCAGGCACCAACAATA | 18 |
| GRMZM2G153233-R | TACCAAGGCAATTCCAAGA | 19 |
| GRMZM2G159399-L | ACACCAAGCCACCAACAAT | 19 |
| GRMZM2G159399-R | TAAGGACAGATGAAGAACCAACAG | 24 |
| GRMZM2G390641-L | GCATTCCAACCTAACTAC | 18 |
| GRMZM2G390641-R | CAGAAGTAATCGTACATCAG | 20 |
| GRMZM2G475882-L | CAGCCAGCAAGAACAATA | 18 |
| GRMZM2G475882-R | GGTCAACAACTCATCATCA | 19 |
| GRMZM5G808366-L | ATAGTGACTTGGCATAGG | 18 |
| GRMZM5G808366-R | TAAGATGGTGAGGTGAAC | 18 |
| GRMZM5G825321-L | GTCCTGTATGTTCCTTCTC | 19 |
| GRMZM5G825321-R | ATCCTCTACTCTCGGTTG | 18 |
| GRMZM5G848945-L | GAGAAGCAACATCTATACG | 19 |
| GRMZM5G848945-R | GAGTAGCCACAATCAATG | 18 |
| GRMZM5G893117-L | GTTTGAAGAAGTTGGGAAGAG | 21 |
| GRMZM5G893117-R | CTAAGCGTCGTCTCACAG | 18 |
| Zm18S-L | AAACGGCTACCACATCCAAG | 20 |
| Zm18S-R | CCTCCAATGGATCCTCGTTA | 20 |
